# Enterovirus D68 VP3 Targets the Interferon Regulatory Factor 7 To Inhibit Type I Interferon Response

**DOI:** 10.1128/spectrum.04138-22

**Published:** 2023-05-01

**Authors:** Jun Kang, Mengqian Huang, Jinyu Li, Keke Zhang, Cheng Zhu, Sihua Liu, Zhenwei Zhou, Tao Wang, Zhiyun Wang

**Affiliations:** a School of Life Sciences, Tianjin University, Tianjin, China; b Institute of Tianjin Key Laboratory of Function and Application of Biological Macromolecular Structures, Tianjin, China; c School of Environmental Science and Engineering, Tianjin University, Tianjin, China; David Geffen School of Medicine at UCLA

**Keywords:** interferon regulatory factor 7, EV-D68, VP3, Toll-like receptor, IFN-I

## Abstract

Enterovirus D68 (EV-D68) is a globally emerging pathogen causing severe respiratory illnesses mainly in children. The protease from EV-D68 could impair type I interferon (IFN-I) production. However, the role of the EV-D68 structural protein in antagonizing host antiviral responses remains largely unknown. We showed that the EV-D68 structural protein VP3 interacted with IFN regulatory factor 7 (IRF7), and this interaction suppressed the phosphorylation and nuclear translocation of IRF7 and then repressed the transcription of IFN. Furthermore, VP3 inhibited the TNF receptor associated factor 6 (TRAF6)-induced ubiquitination of IRF7 by competitive interaction with IRF7. IRF7_Δ305–503_ showed much weaker interaction ability to VP3, and VP3_Δ41–50_ performed weaker interaction ability with IRF7. The VP3 from enterovirus A71 (EV-A71) and coxsackievirus A16 (CV-A16) was also found to interact with the IRF7 protein. These results indicate that the enterovirus structural protein VP3 plays a pivotal role in subverting host innate immune responses and may be a potential target for antiviral drug research.

**IMPORTANCE** EV-D68 is a globally emerging pathogen that causes severe respiratory illnesses. Here, we report that EV-D68 inhibits innate immune responses by targeting IRF7. Further investigations revealed that the structural protein VP3 inhibited the TRAF6-induced ubiquitination of IRF7 by competitive interaction with IRF7. These results indicate that the control of IRF7 by VP3 may be a mechanism by which EV-D68 represses IFN-I production.

## INTRODUCTION

The human enterovirus D68 (EV-D68) is a positive single-stranded RNA virus (1), belonging to the family *Picornaviridae*, genus *Enterovirus*, and species *Enterovirus D* (1, 2). EV-D68 possesses a genome of approximately 7.4 kb in size, encoding four structural proteins, VP1, VP2, VP3, and VP4, and seven nonstructural proteins, 2A, 2B, 2C, 3A, 3B, 3C, and 3D (3, 4). The VP1 protein, which contains eight beta strands linked by seven loop structures, is commonly used for virus classification and detecting newly emerging strains (5). EV-D68 was first isolated in California, USA, in 1962, and caused severe respiratory diseases, such as bronchitis and pneumonia (1, 2). Only twenty-six cases of EV-D68 were reported during 1970 to 2005, according to the statistics of the U.S. National Enterovirus Surveillance System (6). However, in 2014, the outbreak of EV-D68 in the United States caused 1,153 infection cases with severe respiratory symptoms (6, 7). In 2018, a 6-year-old boy was diagnosed with acute flaccid myelitis (AFM) caused by EV-D68 infection, which was the first EV-D68 infection-induced AFM case in mainland China (8). This case once again raised public concerns about the pathogenicity of EV-D68.

The type I interferon (IFN-I) signaling pathway plays a pivotal role in the response of innate immunity during the early stage of viral infection (9, 10). Pathogen recognition mediated by retinoic acid-inducible gene I (RIG-I), melanoma differentiation-associated gene 5 (MDA5), and Toll-like receptors (TLRs) is the major strategy used by the host to recognize viruses (11–13). Unlike TLRs, two cytoplasmic viral RNA sensors, RIG-I and MDA5 (14–17), function by recognizing different viral RNA and then transmit signals to a downstream mitochondrial antiviral signaling (MAVS) protein (18, 19). The virus signal, which is also mediated by tumor necrosis factor receptor-associated factor (TRAF) family members (TRAF3 and TRAF6), then activates the nuclear factor kappa B (NF-κB) signaling pathway and IFN pathway (20). The interferon regulatory factor (IRF) is an essential element to regulate the expression of interferon. Two members of this family, IRF3 and IRF7, have been identified as critical transcription factors of virus-mediated production of IFN-I (IFN-α/β). IRF3 and IRF7 phosphorylation, dimerization, nuclear import, and interaction with transcriptional coactivators activates a robust response comprised of a broader spectrum of IFN isotypes (21, 22). It has been reported that TRAF6 mediates the K63-linked ubiquitination of IRF7, and this process is required for the phosphorylation and activation of IRF7 to induce IFN promoters (23).

According to our previous work, EV-D68 2A^pro^ functions in inhibiting antiviral type I interferon responses by cleaving TRAF3, which is the pivotal protein for type I interferon production (24). The 3C^pro^ of EV-D68 cleaves TRIF3, and IRF7 thus inhibits interferon production and suppresses the host innate immune response (25–27). EV-D68 overexpresses its minireplicon to antagonize intracellular stress granules (SGs), in which SG marker proteins Ras GTPase-activating protein-binding protein 1 (G3BP1), T cell intracellular antigen 1 (TIA1), and human antigen R (HUR) could chelate a specific site of the 3′ untranslated region (3′ UTR) of EV-D68 to inhibit viral replication (4). However, whether any of the EV-D68 structural proteins are involved in the host innate immune system is unclear.

In this work, we focus on how the enterovirus structural protein VP3 inhibits the production of IFN-I. We provide evidence that the EV-D68 structural protein VP3 interacts with IRF7, which results in the suppression of phosphorylation and nuclear import of IRF7. Furthermore, we show that VP3 inhibits the TRAF6-mediated K63-linked ubiquitination of IRF7 and blocks the activation of IRF7, resulting in the downregulation of IFN-I production. The interaction between VP3 from EV-A71 or CV-A16 and IRF7 is also characterized.

## RESULTS

### EV-D68 VP3 suppresses Sendai virus-induced IFN-β activation.

Innate immunity is the host's first line of defense against virus infection. It has been reported that nonstructural proteases of EV-D68 inhibit the production of IFN-I and enable the virus to escape the host's innate immunity (1, 25, 27, 28).

**FIG 1:**
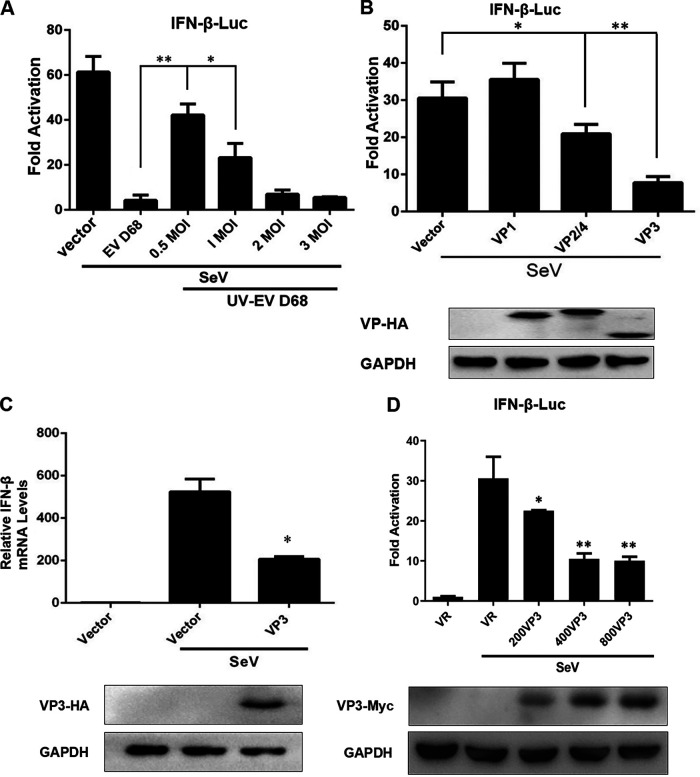
EV-D68 VP3 suppresses virus-induced IFN-β activation. (A) The UV-inactivated EV-D68 virus dose-dependently inhibited the IFN-β promoter induced by SeV infection. RD cells were treated with an increasing dose of UV-EV-D68 (MOI = 0.5, 1, 2, 3) for 24 h. The positive control group was cells infected with active EV-D68 (MOI = 0.5). HEK293T cells were transfected with 200 ng of the reporter plasmid IFN-β-Luc and 2 ng of pRLSV40 for 24 h. After transfection, the cells were treated with SeV (20 HA/mL) or UV-EV D68 (MOI = 0.5, 1, 2, 3) for 24 h, and the cell lysates were subjected to a luciferase assay. (B) Detection of anti-IFN-I responses by viral structural proteins. HEK293T cells were transfected with 200 ng of the reporter plasmid IFN-β-Luc, 2 ng of pRL-SV40, and 600 ng of VP1, VP2/4, or VP3. Twenty-four hours later, the cells were treated with medium or SeV (20 HA/mL) for 16 h, the cell lysates were subjected to a luciferase assay, and the expression of plasmid was confirmed by Western blotting. (C) The treatment of EV-D68 VP3 inhibits IFN-β mRNA synthesis. HEK293T cells were transfected with 600 ng VP3-HA or empty vector. Twenty-four hours after transfection, cells were infected with SeV (20 HA/mL) for 16 h. Total RNA was extracted, and the expression of IFN-β mRNA was determined by quantitative PCR (qPCR) assay. Results are expressed as IFN-β mRNA levels relative to GAPDH RNA levels. Cell lysates were subjected to Western blotting with antibodies against anti-HA and GAPDH. (D) VP3 dose-dependently inhibits SeV-induced IFN-β promoter activity. HEK293T cells were transfected with an increasing dose of VP3 (200 ng, 400 g, 600 ng, and 800 ng). Twenty-four hours later, the cells were treated with medium or SeV (20 HA/mL) for 16 h, the cell lysates were subjected to a luciferase assay, and the expression of plasmid was confirmed by Western blotting. A statistically significant difference was determined using SPSS with a two-tailed Student’s unpaired *t* test (*, *P* < 0.05; **, *P* < 0.01; ***, *P* < 0.001; n.s., not significant).

To explore whether the structural protein plays a role similar to that of nonstructural proteins, the UV-inactivated EV-D68 virus was used, as it lost its ability to replicate and does not contain nonstructural proteins. Firstly, the Sendai virus (SeV) infection activated the IFN-β promoter in HEK293T cells, while EV-D68 infection inhibited SeV-induced IFN-β activation at a rate of ~85%. The UV-inactivated EV-D68 dose-dependently inhibited the IFN-β promoter induced by SeV infection (Fig. 1A). This suggested that the EV-D68 structural proteins may cause the inhibition of the SeV infection-induced IFN-β promoter activity.

To determine the effect of EV-D68 structural proteins on the activation of the IFN-β promoter, we evaluated the anti-IFN-I responses from viral structural proteins using a dual-luciferase reporter assay. The results showed that EV-D68 VP3 markedly inhibited SeV-induced IFN-β promoter activity at a rate of ~80% (Fig. 1B, lane 4). VP1 and VP2/4 showed almost no effect or only 25%, respectively (Fig. 1B, lanes 2 and 3). The IFN-β mRNA synthesis was induced by SeV infection and then inhibited by expression of EV-D68 VP3 (Fig. 1C). In addition, VP3 dose-dependently inhibited SeV-induced IFN-β promoter activity (Fig. 1D), which is consistent with previous results (Fig. 1A). It was suggested that the EV-D68 VP3 protein has a more significant suppression effect on the SeV-induced IFN-β promoter activity than VP1, VP2, and VP4.

### EV-D68 VP3 inhibits activation of the IFN-I pathway by targeting IRF7.

**FIG 2:**
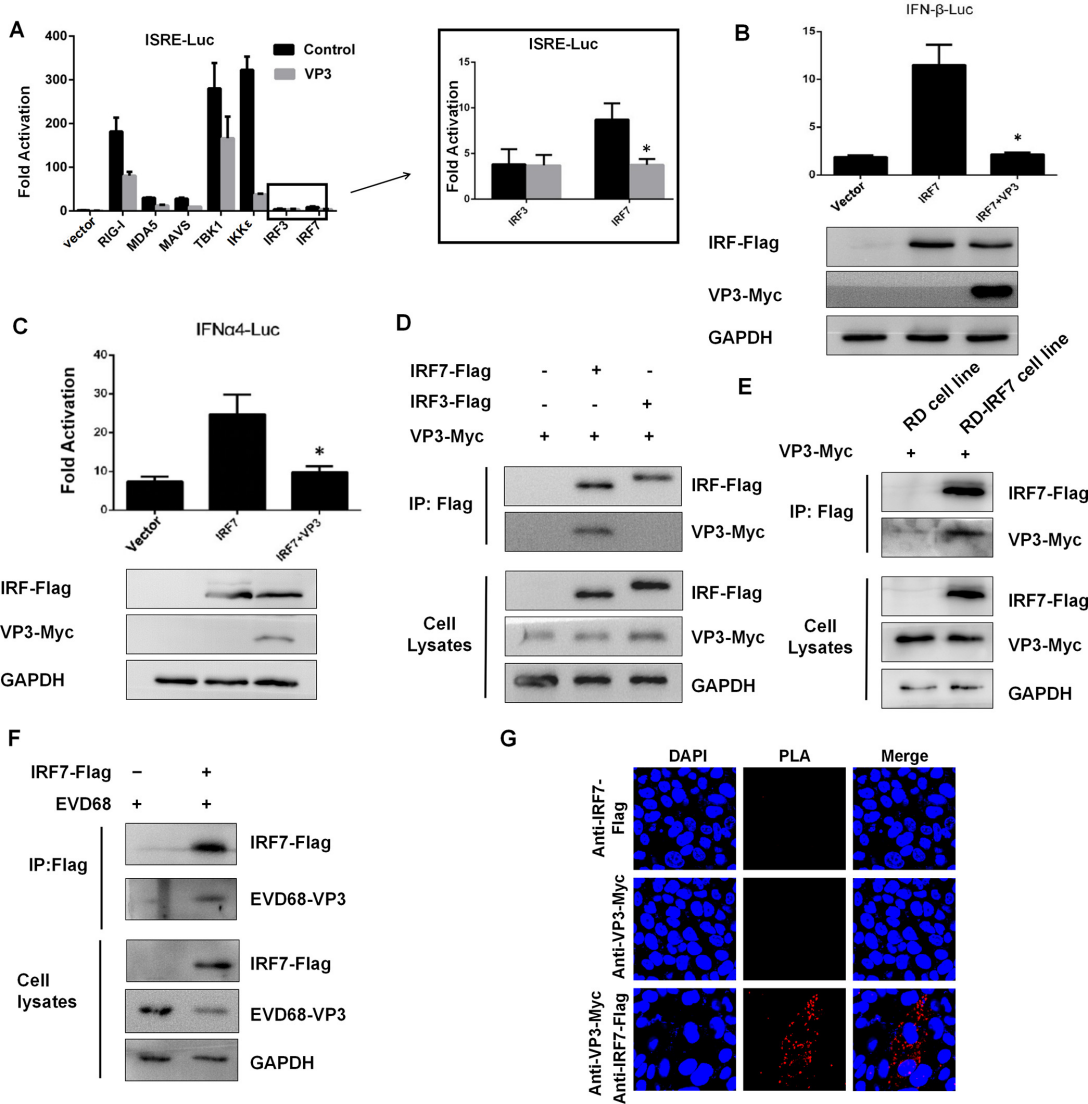
EV-D68 VP3 interacts with IRF7 and inhibits IRF7-mediated activation of the IFN-I pathway. (A) VP3 functions specifically at or downstream of IRF7. HEK293T cells were transfected with 200 ng of ISRE-Luc, 2 ng of pRL-SV40, 600 ng of VP3-Myc, or 600 ng of empty vector (MOCK) and the indicated plasmids, including 200 ng of RIG-I, MDA5, MAVS, TBK1, IKKε, IRF3, or IRF7. Twenty-four hours after transfection, the cell lysates were subjected to a luciferase assay. (B and C) Coexpression of VP3 and IRF7 could inhibit IRF7-induced IFN-I activation. HEK293T cells were transfected with 600 ng VP3-Myc, 300 ng IRF7-Flag, and 200 ng IFN-β-Luc (B) or IFN-α4-Luc (C); 2 ng of pRL-SV40 was used as an internal control. Twenty-four hours after transfection, the cell lysates were subjected to a luciferase assay. (D) VP3 interacts with IRF7 in HEK293T cells. HEK293T cells were transfected with 3 μg VP3-Myc and empty vector or 3 μg IRF7-Flag or IRF3-Flag. The cell lysates were immunoprecipitated (IP) with anti-Flag beads and immunoblotted (IB) with anti-Myc or anti-Flag antibody. (E) VP3 interacts with IRF7 in RD-IRF7-Flag cells. Lane 1 RD cells were transfected with 3 μg VP3-Myc. Lane 1 RD-IRF7-Flag cells were transfected with 3 μg VP3-Myc. The cell lysates were IP with anti-Flag beads and IB with anti-Myc or anti-Flag antibody. (F) EV-D68 virus VP3 protein interacts with IRF7 in HEK293T cells. Lane 1 and 2 HEK293T cells were infected with EV-D68 (MOI = 0.1). Lane 2 HEK293T cells were transfected with 6 μg IRF7-Flag. The cell lysates were IP with anti-Flag beads and IB with anti-EV-D68 VP3 or anti-Flag antibody. (G) The interaction of VP3 and IRF7 in RD-IRF7 cell lines was determined by PLA. The first group used only the RD-IRF7 cell line expressing IRF7, the second group overexpressed the VP3-HA plasmid in RD cells, and the third group overexpressed the VP3-HA plasmid in RD-IRF7 cells. A statistically significant difference was determined using SPSS with a two-tailed Student’s unpaired *t* test (*, *P* < 0.05; **, *P* < 0.01; ***, *P* < 0.001; n.s., not significant).

To elucidate the molecular mechanisms of the VP3-mediated inhibition of SeV-induced IFN production, we used reporter gene assays to identify the cellular target of VP3. There are several elements involved in IFN-I production, including RIG-I, MDA-5, MAVS, TANK binding kinase 1 (TBK1), inhibitor of nuclear factor kappa B kinase subunit epsilon (IKK-ε), IRF3, and IRF7, which act at different stages of the IFN-I signaling pathway (21, 29–31). The results showed that overexpression of VP3 protein inhibited RIG-I, MDA-5, MAVS, IKK-ε, and IRF7-induced ISRE reporter gene activation except for IRF3 (Fig. 2A), suggesting that VP3 targets specifically IRF7 or its downstream players but not IRF3 (Fig. 2A, lanes 7 and 8). Coexpression of VP3 and IRF7 could inhibit the IRF7-induced activation of the IFN-I promoter, including the transcription of IFN-β and IFN-α4, which were 80% and 60% inhibited, respectively (Fig. 2B and C). This was consistent with our previous results as shown in Fig. 1.

To address the potential mechanism of how VP3 suppresses the IRF7-mediated signaling pathway, we examined the interaction between EV-D68 VP3 and IRF7 by transient transfection and coimmunoprecipitation experiments. The results showed that VP3 specifically interacts with IRF7 but not with IRF3 in HEK293T cells (Fig. 2D, lanes 2 and 3). Since the expression of IRF7 is low in HEK293T and RD cells, the RD cell line stably expressing IRF7 named “RD-IRF7” was constructed. Coimmunoprecipitation assay showed that the endogenously expressed IRF7 strongly interacted with VP3 (Fig. 2E). After transmutation of IRF7 protein and infection with EV-D68 virus, coimmunoprecipitation showed that IRF7 protein could enrich VP3 protein of EV-D68 (Fig. 2F). We further validated the interaction relationship between VP3 and IRF7 using proximity ligation assay (PLA). The results were as follows, overexpression of VP3 in RD-IRF7 cells produced distinct red fluorescent spots, each representing an interaction between IRF7 and VP3 (Fig. 2G). These results demonstrated that EV-D68 VP3 inhibits the activation of the IFN-I pathway by targeting IRF7.

### VP3 inhibits SeV-induced IRF7 phosphorylation and nuclear importing.

**FIG 3:**
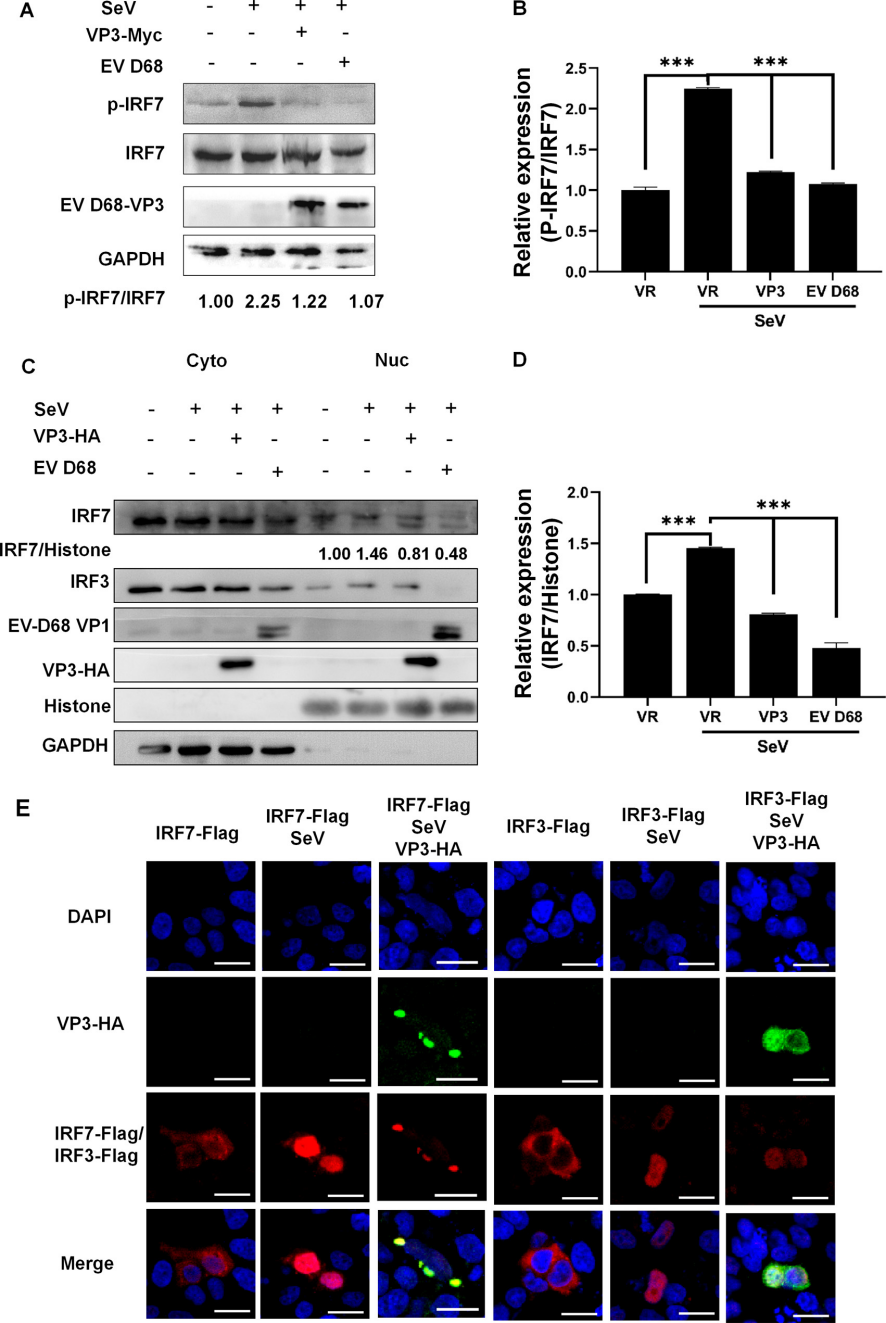
VP3 inhibits SeV-induced IRF7 phosphorylation and nuclear import. (A) p-IRF7 is suppressed by VP3 plasmid treatment or EV-D68 (MOI = 0.1) infection in RD-IRF7 cells. RD-IRF7 cells were transfected with 1 μg VP3-Myc. Twenty-four hours later, cells were infected with SeV (20 HA/mL) for 16 h. Then, the cell lysates were subjected to Western blotting with p-IRF7, IRF7, EVD68-VP3, and GAPDH antibodies. (B) Quantification of the relative expression of P-IRF7/IRF7 in panel A. (C) The endogenous IRF7 expression level was detected. A significant decrease in IRF7 is observed in the nucleus. Lane 3 HEK293T cells were transfected with 1 μg VP3-Myc. Lane 4 HEK293T cells were infected with EV-D68 (MOI = 0.1). Twenty-four hours after transfection, cells were infected with SeV (20 HA/mL) for 16 h. The nuclear and cytoplasmic proteins were separated. Cell lysates were subjected to Western blotting with the following antibodies, anti-IRF7, anti-IRF3, anti-Myc, anti-EV-D68 VP1 and GAPDH. (D) Quantification of the relative expression of IRF7/histone in panel C. (E) EV-D68 VP3 inhibits the nuclear translocation of IRF7 after SeV stimulation in HeLa cells. HeLa cells were seeded on glass coverslips overnight and transfected with 300 ng IRF7/IRF3-Flag or 500 ng VP3-HA or both plasmids, 24 h after transfection, cells were infected with SeV (20 HA/mL) for 16 h. Then, the cells were analyzed using immunofluorescence staining with anti-HA (green), anti-Flag (red), and DAPI (blue) and microscopy. A statistically significant difference was determined using SPSS with a two-tailed Student’s unpaired *t* test (*, *P* < 0.05; **, *P* < 0.01; ***, *P* <0.001; n.s., not significant).

In the process of responding to EV-D68 infection, cytosolic RIG-I and MDA5 initiate antiviral signaling and then recruit and activate downstream molecules. IFN-I transcriptional expression requires IRF7 phosphorylation, dimerization, and nuclear import. To confirm the inhibition effect of EV-D68 VP3 on IFN-I production, we examined whether VP3 could influence the phosphorylation status of IRF7. The results showed that the SeV-triggered accumulation of endogenous p-IRF7 was inhibited by both exogenous VP3 plasmid and EV-D68 infection in RD-IRF7 cells (Fig. 3A). Both transient transfection of VP3 and infection with EV-D68 virus could reduce the p-IRF7 protein expression by 50% (Fig. 3B). Besides, with EV-D68 treatment, the nuclear localized endogenous IRF7 expressed at a much lower level than that in non-virus-treated cells, indicating that the EV-D68 inhibited the nuclear importing of endogenous IRF7 (Fig. 3C and D). In addition, the immune fluorescence experiment was used to analyze the nuclear import level of the IRF7 protein. It was found that EV-D68 VP3 inhibited the nuclear translocation of IRF7 but not with IRF3 with SeV stimulation in HeLa cells (Fig. 3E, row 3, columns 3 and 6). Two hundred cells were counted for each experimental condition, and data analysis showed that in the group transfected with only IRF7-Flag, IRF7 protein was imported into the nucleus in ~10% of cells. Simultaneously, in the group transfected with IRF7-Flag associated with SeV stimulation, IRF7 protein was imported into the nucleus in ~80% of cells. However, in the cell group transfected with both VP3 and IRF7 plasmids associated with SeV stimulation, the IRF7 protein entered the nucleus in ~25% of the cells (see Fig. S1A and B in the supplemental material). These results indicated that VP3 significantly inhibits the SeV-induced IRF7 phosphorylation and nuclear translocation.

### VP3 inhibits TRAF6-induced ubiquitination level and the transactivation ability of IRF7.

**FIG 4:**
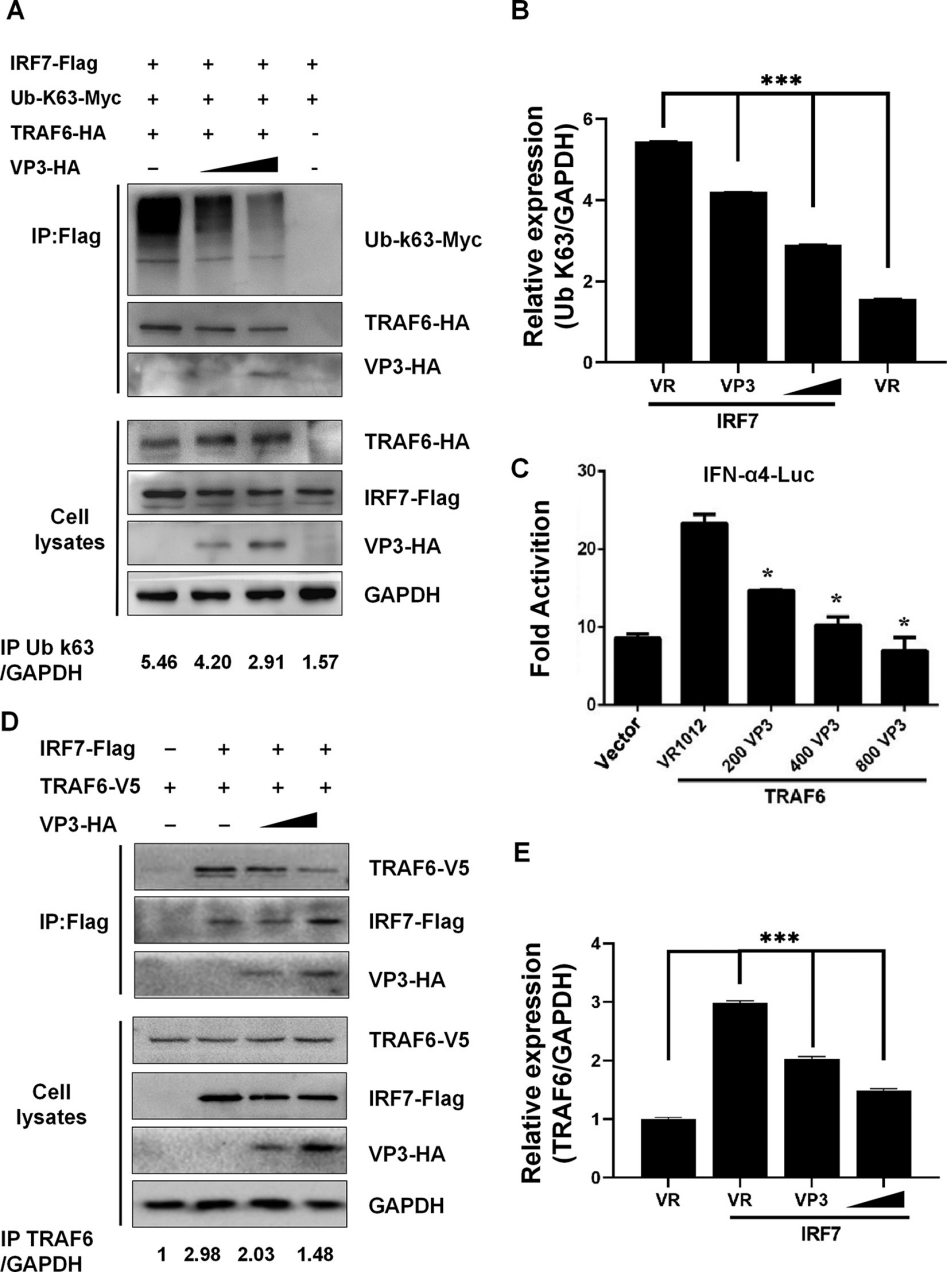
VP3 inhibits the TRAF6-induced ubiquitination level and the transactivation ability of IRF7. (A) EV-D68 VP3 disrupts the interaction of TRAF6 and IRF7 in a dose-dependent way. HEK293T cells were transfected with 2 μg IRF7-Flag, 2 μg Ub-K63-Myc, 2 μg TRAF6-HA or empty vector, and 2 μg VP3-HA or empty vector. Cell lysates were immunoprecipitated with anti-Flag beads followed by immunoblots using HA, Flag, Myc, and GAPDH antibodies. (B) Quantification of the relative expression of K63/GAPDH in panel A. (C) The TRAF6-induced IRF7-dependent transcription level of IFN-α4-Luc was suppressed. HEK293T cells were transfected with VP3-HA (200 ng, 400 ng, 800 ng), 150 ng of TRAF6-V5, and 200 ng of IFN-α4-Luc; 2 ng of pRL-SV40 was used as an internal control. Twenty-four hours after transfection, the cell lysates were subjected to a luciferase assay. (D) Expression of VP3 protein dose-dependently inhibits TRAF6 induced ubiquitination of IRF7. HEK293T cells were transfected with 1.5 μg IRF7-Flag, 2.5 μg VP3-HA, and 600 ng TRAF6-V5. Cell lysates were IP with anti-Flag beads. Immunoprecipitates and aliquots of cell lysates were subjected to Western blotting with HA, Flag, V5, and GAPDH antibodies. (E) Quantification of the relative expression of TRAF6/GAPDH in panel D. A statistically significant difference was determined using SPSS with a two-tailed Student’s unpaired *t* test (*, *P* < 0.05; **, *P* < 0.01; ***, *P* < 0.001; n.s., not significant).

It has been reported that TRAF6 mediating the K63-linked ubiquitination of IRF7 is required for the phosphorylation and activation of IRF7 to induce IFN responses (23). This phenomenon inspired us to consider whether VP3 could affect the ubiquitination of IRF7. We cotransfected TRAF6-V5, Ub-K63, and IRF7-Flag in HEK293 cells. It was observed that the ubiquitination level of IRF7 was strongly induced by TRAF6 overexpression. Immunoblotting analysis showed the exogenous expression of VP3 inhibited TRAF6-induced ubiquitination of IRF7 in a dose-dependent manner (Fig. 4A and B). Similarly, the reporter gene assays showed that in cells that expressed VP3 protein, the TRAF6-induced IRF7-dependent transcription level of *IFN-α4-Luc* was suppressed (Fig. 4C, lanes 2, 3, and 4).

Considering TRAF6 directly binds and ubiquitinates the transcription factor IRF7, we performed a competitive coimmunoprecipitation assay in HEK293T cells transfected with exogenous VP3-HA, IRF7-Flag, and the TRAF6-V5 plasmid. Western blot analysis showed that the VP3 treatment reduced TRAF6 expression level in a dose-dependent way in cells coexpressed with IRF7-Flag (Fig. 4D and E, lanes 2, 3, and 4). This indicated that EV-D68 VP3 could disrupt the interaction of TRAF6 and IRF7.

Taken together, these results revealed that VP3 inhibits the TRAF6-induced ubiquitination level and transcriptional activation ability of IRF7 by disrupting the interaction between TRAF6 and IRF7.

### Identification of VP3 and IRF7 interacting region.

**FIG 5:**
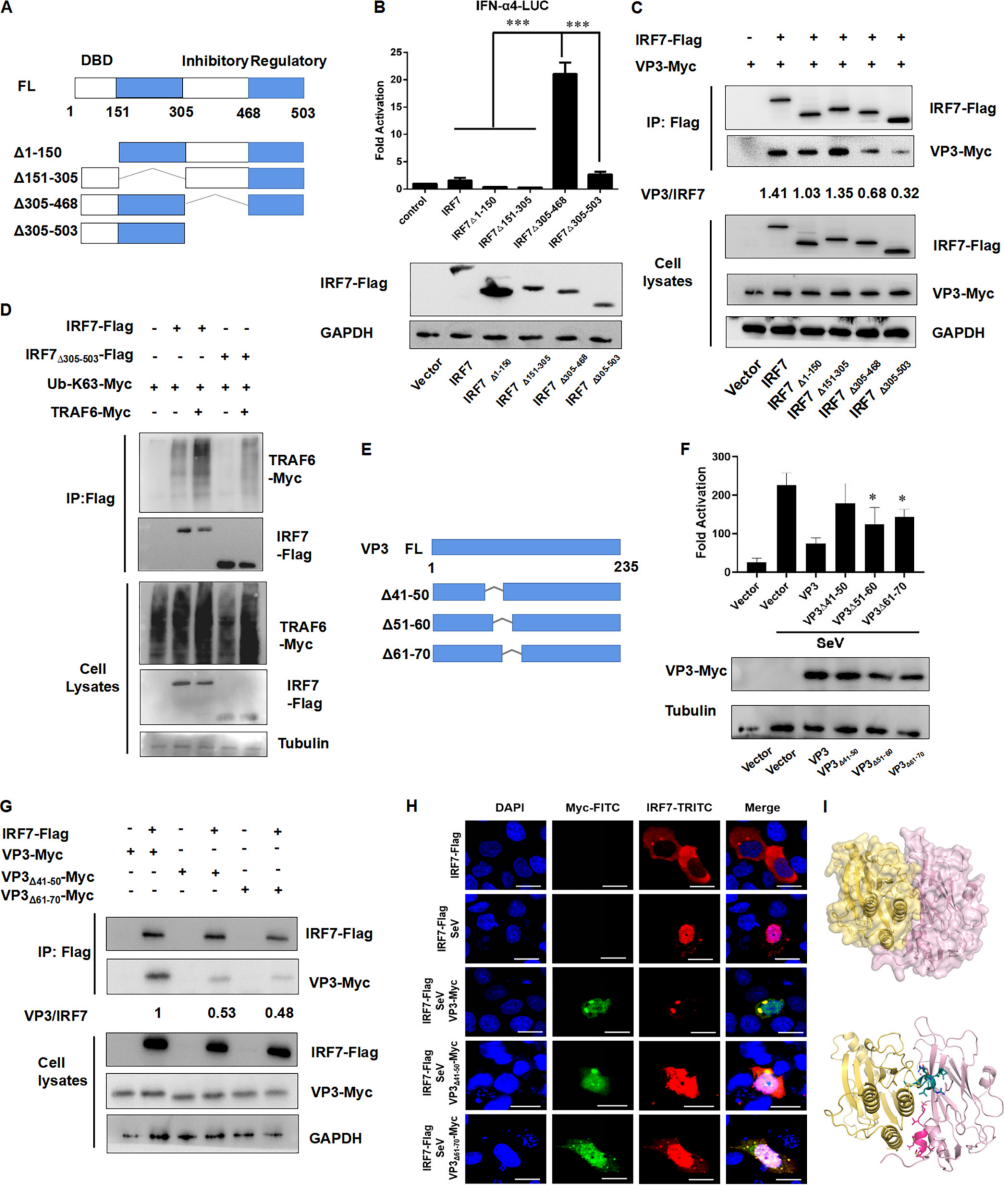
Identification of VP3 and IRF7 interacting region. (A) Four Flag-tagged IRF7-truncated mutants were constructed. (B) Removing 305 to 468 amino acids resulted in a constitutively active form of IRF7. HEK293T cells were transfected with 600 ng of IRF7-truncated mutants, 200 ng of IFN-β-Luc, and 2 ng of pRL-SV40. The cell lysates were subjected to a luciferase assay, and the expression of plasmid was confirmed by Western blotting. (C) IRF7_Δ305–503_ could not be coimmunoprecipitated with VP3. HEK293T cells were cotransfected with 3 μg of VP3-Myc and 3 μg of IRF7-truncated mutants. The cell lysates were immunoprecipitated with anti-Flag and immunoblotted with anti-HA or anti-Flag. (D) TRAF6 binds to the C terminus of IRF7. The 305 to 503 amino acid region of IRF7 is the major binding site of TRAF6 to IRF7 protein to facilitate K63-linked polyubiquitination. HEK293T cells were cotransfected with 2 μg IRF7-Flag or IRF7_Δ305–503_ or empty vector, 2 μg Ub-K63-Myc, and 2 μg TRAF6-Myc or empty vector. The cell lysates were immunoprecipitated with anti-Flag and immunoblotted with anti-Myc or anti-Flag. (E) Three VP3-truncated mutants were constructed. (F) HEK293T cells were transfected with 600 ng VP3, VP3_Δ41-50,_ VP3_Δ51–60,_ VP3_Δ61–70_, and 200 ng of IFN-β-Luc; 2 ng of pRL-SV40 was used as an internal control. Twenty-four hours after transfection, the cell lysates were subjected to a luciferase assay. (G) Wild-type VP3, VP3_Δ61–70_ interacted with IRF7-Flag more strongly than VP3_Δ41–50_. HEK293T cells were cotransfected with 3 μg IRF7-Flag and 3 μg the indicated Myc-tagged plasmids. The cell lysates were immunoprecipitated with anti-Flag and immunoblotted with anti-Myc or anti-Flag. (H) Effects of EV-D68 VP3 truncation mutants on nuclear translocation of IRF7 after SeV stimulation in HeLa cells. HeLa cells were seeded on glass coverslips overnight and transfected with 300 ng IRF7-Flag or 500 ng VP3-Myc or 500 ng VP3_Δ41–50_-Myc or 500 ng VP3_Δ61–70_-Myc; 24 h after transfection, cells were infected with SeV (20 HA/mL) for 16 h. Then, the cells were analyzed using immunofluorescence staining with anti-Myc (green), anti-Flag (red), DAPI (blue), and microscopy. (I) The predicted binding model between VP3 and IRF7. Yellow, aa 305 to 503 of IRF7; pink, VP3; cherry, aa 41 to 50 of VP3, RNMLEMIQVE; green-blue, aa 61 to 70 of VP3, GANGMERLRV.

Structural studies have identified that IRF7 contains the following domains: the conserved N-terminal DNA-binding domain (DBD), IRF-association domain, an autoinhibitory domain, and a signal responding domain. To identify the IRF7 sequence responsible for interacting with VP3, four Flag-tagged IRF7-truncated mutants were constructed as described in Fig. 5A.

The *IFN-a4* promoter activity was detected in HEK293T cells with different IRF7-truncated plasmid treatment. It was found that both IRF7_Δ305–503_ and IRF7_Δ305–468_ enhanced *IFN-a4* promoter activity more strongly than full-length IRF7 did, while IRF7_Δ1–150_ and IRF7_Δ151–305_ did not (Fig. 5B, lane 5). This indicated that the region of amino acids (aa) 305 to 468 of the IRF7 protein is the pivotal region to activate the IFN promoter. Then, all of the IRF7 mutant plasmids were cotransfected into HEK293T cells with Myc-tagged VP3, respectively. With the coimmunoprecipitation assay, we found that IRF7, IRF7_Δ1–150_, and IRF7_Δ151–305_ could be coimmunoprecipitated with VP3 at a normal level, while IRF7_Δ305–468_ and IRF7_Δ305–503_ bound a much lower level of VP3, especially IRF7_Δ305–503_ (Fig. 5C; see also Fig. S2A, lane 6, in the supplemental material), suggesting that the aa 305 to 503 region of IRF7 is essential for the interaction between IRF7 and VP3. Then, we checked whether the TRAF6 binds to the C terminus of IRF7 to facilitate K63-linked polyubiquitination. The results indicated that the aa 305 to 503 region of IRF7 is the major binding site of TRAF6 to IRF7 protein to facilitate K63-linked polyubiquitination (Fig. 5D).

Then, the interacting region of VP3 with IRF7 was examined. VP3-truncated mutants, VP3_Δ41–50_, VP3_Δ51–60_, and VP3_Δ61–70_ were constructed (Fig. 5E). The *IFN-a4* promoter activity was measured in HEK293T cells with wild-type (wt) and truncated VP3 plasmid transfection, respectively. Results showed that the VP3_Δ41–50_ inhibited *IFN-β* promoter activities much weaker than VP3_wt_ and other VP3 mutants did (Fig. 5F). Transfection and coimmunoprecipitation experiments both showed that VP3_wt_, VP3_Δ41–50_, and VP3_Δ61–70_ could still interact with IRF7-Flag, while both of the mutants performed a much weaker interacting ability with IRF7 (Fig. 5G; see also Fig. S2B). We then examined whether the nuclear entry pattern of IRF7 could be interfered with by VP3 treatment using immunofluorescence analysis. The VP3_wt_ and truncated plasmids were cotransfected with IRF7, respectively, and then stimulated with SeV virus. It was found that VP3_Δ41–50_ and VP3_Δ61–70_ did not affect the nuclear entry and function of IRF7, while VP3_wt_ and VP3_Δ51–60_ did (Fig. 5H).

Furthermore, the crystal structure of VP3 (adopted from EV-D68 structure; PDB accession number 4WM7) and the AlphaFold2 predicted model of IFR7 were applied as inputs to HDOCK (32, 33). Top ranking models of VP3-IRF7 complexes were clustered and analyzed based on binding interface properties (Fig. 5I). The yellow sequence was aa 305 to 503 of IRF7, the pink part was full-length VP3, the cherry residue was the N-terminal aa 41 to 50 of VP3, and the green-blue residue represented the aa 61 to 70 of VP3. The close binding interface between aa 305 to 503 IRF7 and aa 41 to 50 or aa 61 to 70 of VP3 was consistent with the coimmunoprecipitated results shown in Fig. 5F.

Combined with the *IFN-a4* promoter activities shown in Fig. 5E, we speculated that the N-terminal amino acid sequence 41 to 50 of VP3 structural protein is the key region for the interaction between IRF7 and VP3.

### The VP3 protein from both CV-A16 and EV-A71 interact with IRF7 and inhibit SeV-induced IRF7-dependent transcriptional activation.

**FIG 6:**
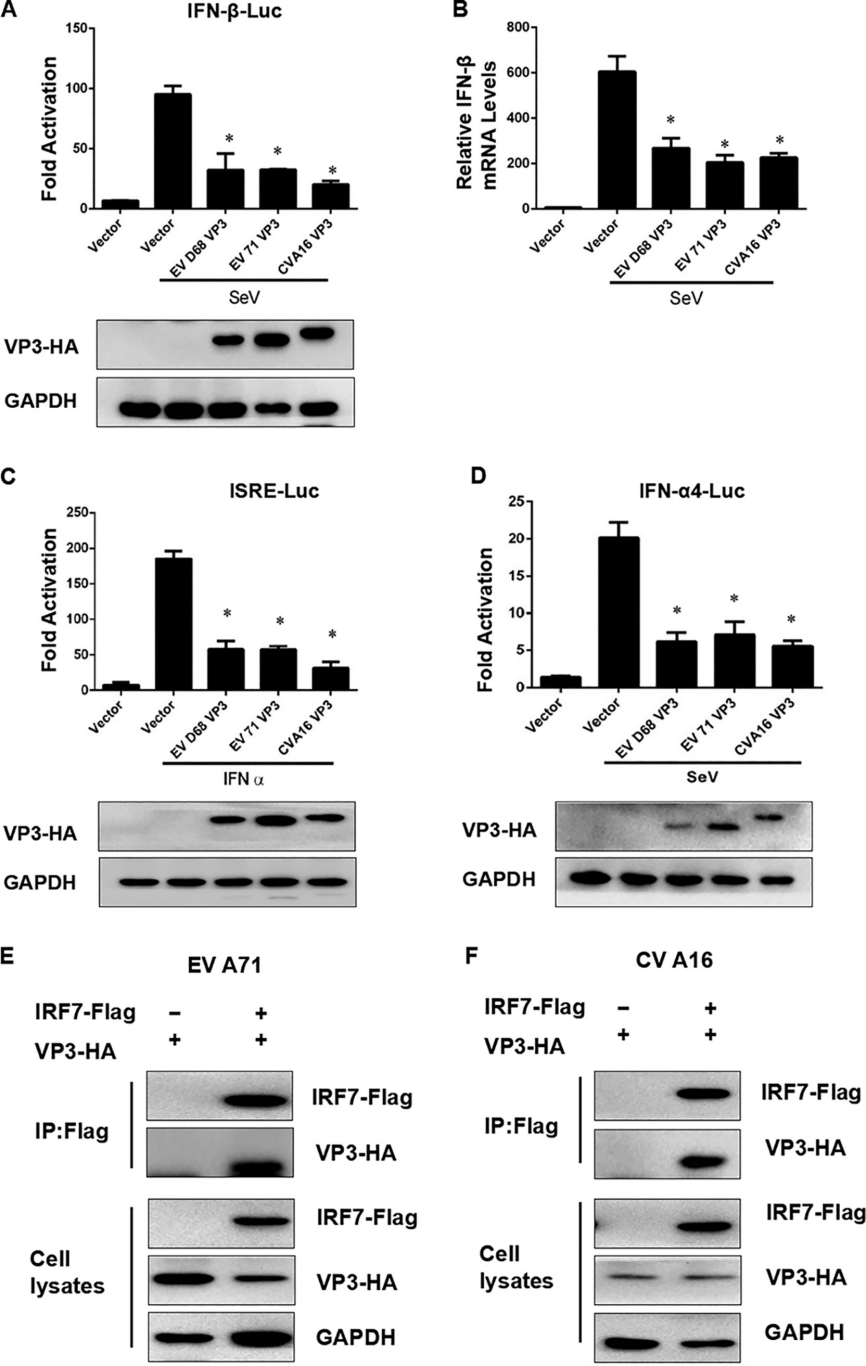
The VP3 proteins of CV-A16 and EV-A71 interact with IRF7. (A) The exogenous expression of VP3 protein from EV-A71 and CV-A16 inhibited SeV-induced IRF7-dependent transcriptional activation. HEK293T cells were transfected with 600 ng VP3-HA, 600 ng EV-A71 VP3-HA, 600 ng CV-A16 VP3-HA, and 200 ng IFN-β-Luc; 2 ng pRL-SV40 was used as an internal control. Twenty-four h after transfection, cell lysates were assayed for promoter-luciferase activities. Cell lysates were subjected to Western blotting with HA and GAPDH antibodies. (B) EV-A71 and CV-A16 VP3 inhibit the SeV-induced production of IFN-β mRNA. HEK293T was transfected with 4 μg IRF7-Flag and 4 μg EV-A71 VP3-HA or CVA16 VP3-há; 48 h after transfection, cell lysates were IP with anti-Flag beads. Immunoprecipitates and aliquots of cell lysates were subjected to Western blotting with HA, Flag, and GAPDH antibodies. (C and D) The exogenous expression of VP3 protein from EV-A71 and CV-A16 inhibits SeV-induced IRF7-dependent transcriptional activation. HEK293T cells were transfected with 600 ng VP3-HA, 600 ng EV-A71 VP3-HA, 600 ng CV-A16 VP3-HA, and 200 ng ISRE-Luc (C) or IFN-α4-Luc (D); 2 ng pRL-SV40 was used as an internal control. Twenty-four hours after transfection, cell lysates were assayed for promoter-luciferase activities. Cell lysates were subjected to Western blotting with HA and GAPDH antibodies. (E) EV A71 VP3 interacts with IRF7 in HEK293T cells. HEK293T cells were transfected with 600 ng VP3-HA and empty vector or 300 ng IRF7-Flag or IRF3-Flag. The cell lysates were IP with anti-Flag beads and IB with anti-HA or anti-Flag antibody. (F) CV A16 VP3 interacts with IRF7 in HEK293T cells. HEK293T cells were transfected with 600 ng VP3-HA and empty vector or 300 ng IRF7-Flag or IRF3-Flag. The cell lysates were IP with anti-Flag beads and IB with anti-HA or anti-Flag antibody.

To investigate whether the function of VP3 protein is conserved, we selected another two enteroviruses, enterovirus A71 (EV-A71) and coxsackievirus A16 (CV-A16), to verify our conjecture. The reporter gene assays showed that both of the exogenous expression of VP3 protein from EV-A71 and CV-A16 inhibited SeV-induced IRF7-dependent transcriptional activation, including IFN-β, ISRE, and IFN-α4 A promoter activation (Fig. 6A to D). The EV-A71 and CV-A16 VP3 also inhibited the SeV-induced production of IFN-β mRNA (Fig. 6B, lanes 4 and 5). To verify whether VP3 protein 41 to 50 and 61 to 70 aa are conserved in other viruses, we constructed four kinds of truncated VP3; they were EV-A71-VP3_Δ41-50_, EV-A71-VP3_Δ61-70_, CV-A16-VP3_Δ41-50_, and CV-A16-VP3_Δ61-70_. Next, we verified whether the four truncated VP3 could inhibit interferon expression. The results showed that with either EV-A71-VP3_Δ41-50_ or CV-A16-VP3_Δ41-50_ treatment, the expression of interferon could not be further inhibited, while the wild-type VP3 still could (see Fig. S3A and B in the supplemental material). After deletion of the 61 to 70 aa region of VP3 of EV-A71 and CV-A16, VP3 could still inhibit the expression of interferon. This result indicated that the 41 to 50 aa region but not the 61 to 70 aa region of VP3 in EV-A71 and CV-A16, plays a pivotal role in inhibiting the expression of interferon.

Then, we tested whether there was a direct interaction between VP3 from EV-A71 or CV-A16 and IRF7. Western blotting showed that the exogenous VP3 protein of the two viruses could interact with IRF7 (Fig. 6E and F), indicating that the VP3 enterovirus structural proteins consistently help the virus escape from the host’s innate immunity by inhibiting IFN-I through interactions with IRF7.

## DISCUSSION

**FIG 7:**
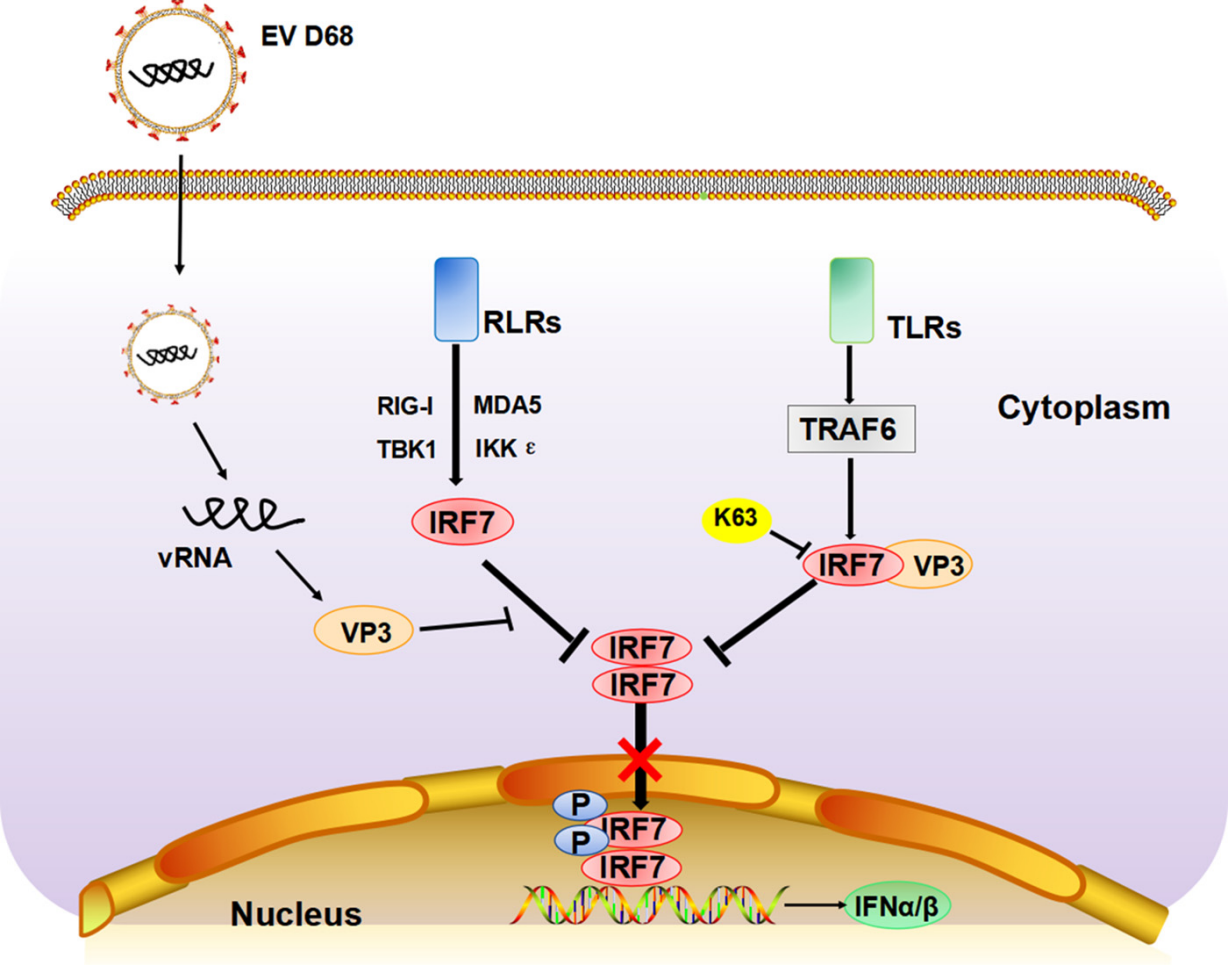
The molecular model of IRF 7 targeting by EV-D68 VP3 to inhibit type I interferon response. The EV-D68 structural protein VP3 interacts with IRF7, resulting in the inhibition of virus-induced phosphorylation and nuclear translocation of IRF7 and suppression of downstream IFN production. EV-D68 VP3 also disturbs the TRAF6-triggered K63-ubiquitination of IRF7, thus inhibiting the phosphorylation of IRF7 and the following *IFN* promoter activation and *IFN* expression.

Viral infection causes an immediate innate immune response, which can recognize viral components and trigger downstream signal transduction, causing the expression of IFN. The constitutive expression of IFN is the primary defense against viral pathogens, which is essential for maintaining immune homeostasis (34). Previous studies on enterovirus have mostly focused on the function of the nonstructural protease, such as 2A^pro^ and 3C^pro^, in cleaving critical elements involved in the host innate immune signaling pathway (35–38). However, little is known on how the structural proteins affect the transduction of IFN signaling. In this work, we showed for the first time that the EV-D68 structural protein VP3 interacts with IRF7, resulting in the inhibition of virus-induced phosphorylation and nuclear translocation of IRF7 and suppression of downstream IFN production. Although this is inconsistent with the findings of a previously reported article. It was that the structural protein of EV-A71 into the pEGFPC1 vector formed a VP3 protein with a green fluorescent protein (GFP) fusion tag and found that the VP3-GFP protein did not affect interferon. We speculated that GFP protein has a large amino acid content and may affect its protein structure after forming fusion protein with VP3, so they did not find the phenomenon that VP3 affects interferon (26). Shu et al. also reported that VP3, VP0, 2B, 2C, 3A, and other proteins of the foot-and-mouth disease virus (FMDV) had inhibitory effects on SeV-activated interference (26, 39, 40). EV-D68 VP3 also disturbs the process that TRAF6 triggered K63 ubiquitination to IRF7, thus inhibiting IFN promoter activation and the following IFN expression. In addition, this mechanism seems to be conserved in enteroviruses, as the VP3 proteins of EV-A71 and CV-A16 were shown to target IRF7 (Fig. 7). This IFN inhibitory mechanism regulated by VP3 protein in our current study provides new insight into how enteroviruses evade innate host immunity with the help of a structural protein.

We discovered that the enteroviruses regulate the activity of IRF7 to fully escape innate immunity. IRF7 is a master regulator of the expression of the interferon-related genes in the inflammation response (41). IRF7 undergoes various posttranslational modifications (PTMs), such as phosphorylation, ubiquitination, and sumoylation, and then is imported to the nucleus and positively regulates IFN transcription (42, 43). In our study, we demonstrated that VP3 inhibits the phosphorylation and nuclear import of IRF7, which affects the transcription of interferon-related genes and weakens the host's antiviral response (Fig. 3).

Phosphorylation and ubiquitination modification of transcriptional factors are major mechanisms that regulate transcriptional activation (41, 44). This type of regulatory mechanism is commonly involved not only in immune responses but also in other biological phenomena. Several studies have shown that viruses prevent the normal process of antiviral response by affecting the activation of transcription factors by the action of viral protein or indirectly by the action of induced proteins from the host. It has been reported that N-Myc and STATs interactor (Nmi) could inhibit virus-triggered IFN-I production by increasing the level of ubiquitination and subsequent degradation of IRF7 (45). TRAF6 is a member of the TRAF protein family. Meanwhile, K63 polyubiquitination of IRF7 mediated by TRAF6 can promote IRF7 activation and further activate IFN-related gene transcription (46–49). Our data revealed that VP3 competitively interacts with IRF7, and this interaction appears to block the binding of TRAF6 and IRF7, resulting in the inhibition of the ubiquitination level of IRF7 (Fig. 4D and Fig. 7). These data enrich our understanding of enterovirus structural proteins participating in the ubiquitination process of IRF7 and inhibiting IFN signaling. It was shown that the structural protein VP3 of foot-and-mouth disease virus (FMDV) inhibits the IFN-β signaling pathway by interacting with MAVS and disrupting its mRNA (40). Our results indicate that the function of enterovirus VP3 protein is broad-spectrum in EV-A71 and CV-A16 and that VP3 from EV-A71 and CV-A16 could target IRF7 to antagonize interferon signaling pathways (Fig. 6). The data confirm our hypothesis that the enterovirus structural protein VP3 has a consistent inhibition of interferon response. These observations also support the view that enteroviruses share common mechanisms to inhibit IFN induction. The contribution of VP3 protein from different enteroviruses in viral replication awaits further investigation.

The interacting region between VP3 and IRF7 was also identified (Fig. 5). We found that both N terminal aa 41 to 50 and aa 61 to 70 of the VP3 protein interact with the aa 305 to 503 region of IRF7. However, aa 41 to 50 performed stronger interaction with IRF7 than aa 61 to 70 did. In the work of Li et al., VP3 was found to interact with the MAVS at the C-terminal aa 111 to 220, which inhibits the expression of MAVS by disrupting the mRNA (40). Another work by Ning et al. reported that the last three C-terminal lysine sites (444, 446, and 452) of human IRF7 variant A are essential for the ubiquitination-mediated activation of IRF7 (23). Our work has expanded our understanding of the functional interacting regions of VP3 and IRF7, providing new evidence for the mechanism of viral structural protein escaping from host innate immunity.

It makes sense that viruses use structural proteins first to inhibit the host interferon signaling pathways and help virus replication. This phenomenon is not a kind of functional redundance where both the structural and nonstructural proteins of the virus could inhibit the innate immune system. Recently, it was found that the EV-A71 VP1 interacts with the autophagy-related protein PHB2, which results in highjacking of host cell autophagy to promote EV-A71 replication (50). In another study, the DnaJ heat shock protein family (Hsp40) member A3 (DNAJA3), a novel FMDV VP1-interacting protein, was reported to inhibit FMDV replication by inducing lysosomal degradation of VP1 and attenuating its antagonistic role in the IFN-β signaling pathway (51). Platelet factor 4 (PF4) prevents virus entry into host cells through its interaction with the VP3 protein of EV-A71/CV-A16 and with SCARB2 receptor-mediated endocytosis of EV-A71 and CV-A16 (52). Based on our results, we suspect that when viruses invade host cells, structural proteins first make contact with the cytoplasmic environment and react before the nonstructural protein to counteract the host antiviral response.

SGs represent a highly dynamic cytoplasmic lesion that is critical for cell survival during viral infection. VP3 overexpression in Fig. 3E and IRF7 overexpression in Fig. 5H appear to trigger the formation of cytoplasmic foci very similar to stress granules. Studies have shown that IRF7 can enter SGs (53), so we speculate whether stress granule markers are also present in these aggregates of VP3 and IRF7. After the external transfer of VP3 and IRF7, we used SGs marker protein antibodies G3BP1 and TIA1 for coincubation. No SG copolymerization particles, such as those produced after the influence of Ars, were found (see Fig. S4 in the supplemental material). Therefore, we have no evidence to prove that the copolymer of VP3 and IRF7 is related to SGs at present. Still, we will continue to conduct a series of verifications in the future.

In this study, we report a novel mechanism by which an enterovirus antagonizes the cell’s innate immunity. The structural EV-D68 VP3 protein interferes with IRF7 phosphorylation and nuclear translocation to inhibit IFN production and also inhibit the TRAF6-mediated K63 ubiquitination of IRF7. These data strongly suggest that structural proteins play a critical role in inhibiting innate immunity during enterovirus infection. The enterovirus structural protein VP3 is a potential target for new antiviral drug research and development in the future.

## MATERIALS AND METHODS

### Cells culture and transfection.

HEK293T, rhabdomyosarcoma (RD), and HeLa cells were cultured in high-glucose Dulbecco's modified Eagle's medium (DMEM) (Gibco; C11995500BT). All of the media were supplemented with 10% fetal bovine serum (FBS) (purchased from Ausbian; catalog number VS500T) and 1% penicillin/streptomycin (HyClone) (purchased from the United States) under humidified conditions with 5% CO_2_ at 37°C. For transfection, plasmids were transfected using Lipofectamine 2000 (Invitrogen; lot number 1952312) or polyethyleneimine (PEI) (purchase from Sigma) according to the instructions. The three plasmids Phage-IRF7, psPAX2, and pMD2G were packaged and formed lentivirus. The stable IRF7-expressing RD cell lines were then harvested by the lentivirus infection.

### Viruses and infection.

The EV-D68 Fermon strain (GenBank accession number KU844179.1) was a gift from Xiaofang Yu (Johns Hopkins University). The Sendai virus (SeV) BB1 strain was kindly provided by Lishu Zheng (China Centers for Disease Control and Prevention). For viral infection, cells were infected with EV-D68 with different multiplicities of infection (MOI) in FBS-free medium at 37°C. After 2 h, the supernatant was discarded, the cells were washed three times with phosphate-buffered saline (PBS) (purchased from Gibco), and cells were supplemented with fresh medium.

### Plasmid constructs and reagents.

Plasmid IRF7-Flag was a gift from Niu Junqi (Jilin University). Plasmids IRF3-Flag, IRF7-Flag mutants, EV-D68 VP3-HA, EV-D68 VP3-Myc, EV-D68 VP3-Myc mutants, EV71 VP3-HA, CA16 VP3-HA, TRAF6-Flag, and TRAF6-V5 were constructed in the lab. TRAF6-Flag was bought from Miaoling Biology Company. Wang Jianwei, from the Institute of Pathogenic Biology, Chinese Academy of Medical Sciences, kindly provided pGL3-IFN-α4-luc, ubiquitinating K63, IFN-β-luc, and ISRE-luc. Xiaofang Yu kindly provided VR1012. PcDNA3.0 and pRL-SV40 were purchased from Promega. All variants were confirmed by subsequent sequencing.

Antibodies against HA and Flag were purchased from ABclonal (catalog numbers AE008 and AE005, respectively); antibodies against Myc were purchased from Proteintech (catalog number 16286-1-AP); antibodies against EV-D68 VP3 were purchased from Gene Tex (catalog number GTX132315). Rabbit antibodies against IRF3, IRF7, and p-IRF7 were purchased from Cell Signaling Technology (lot numbers 11904P and 4920S, respectively). Goat anti-mouse IgG, goat anti-rabbit IgG, anti-tubulin monoclonal antibody, and anti-GAPDH (glyceraldehyde-3-phosphate dehydrogenase) monoclonal antibody were purchased from Tianjin Sungene Biotech Co., Ltd. Anti-HA affinity matrix (catalog number 11815016001) was purchased from Roche, and anti-Flag affinity matrix (catalog number A2220) was purchased from Sigma.

### Dual-luciferase reporter system.

HEK293T cells were seeded in 24-well plates. The cells were then transfected at a density of 70% to 80% with a control plasmid or a corresponding plasmid along with pGL3-IFN-β-luc, ISRE-luc, and pRL-SV40 using UltraFection 3.0 (4A Biotech Co., Ltd, Beijing, China). All dual-luciferase reporter experiments were performed using UltraFection 3.0, highly efficient transfection reagents from 4A Biotech, except for the experiments with IRF7, in which PEI was used. Twenty-four hours after transfection, cells were infected with SeV (20 HA/mL) and EV-D68 (MOI = 0.1) for 16 h. Cell lysates were used to determine luciferase activity using a dual-luciferase reporter system (MicroWIN; Siemens) according to the manufacturer's instructions. A dual-luciferase reporter assay system was used to analyze the luciferase activity in the lysates.

### Nuclear extracts.

Cells were harvested and washed with PBS. A 5-fold volume of the fresh, prechilled cytoplasmic lysate (0.5-mM dithiothreitol [DTT], 1 mM cocktail) was added. The cells were resuspended and left on ice for 15 min, repeatedly pipetted, and centrifuged at 6,000 × *g* for 20 min at 4°C. The cytoplasms were in the supernatant and the nuclei in the pellet. Fifty microliters of radioimmunoprecipitation assay (RIPA) were added to precipitate and pipetted repeatedly, and the samples then were left on ice for 20 min to produce the nuclear lysates.

### Viral RNA extraction, reverse transcription, and quantitative real-time reverse transcription-PCR.

QIAzol lysis reagent (catalog number 79306; Qiagen) was used to extract total intracellular RNA. RNA reverse transcription was performed using TransScript first-strand cDNA synthesis SuperMix kit (catalog number AE301). Quantitative real-time PCR was conducted using the Transgene reverse transcription-PCR (RT-PCR)-related kit (SYBR green) to detect changes in mRNA levels, and the levels of mRNAs were normalized to glyceraldehyde-3-phosphate dehydrogenase (GAPDH) mRNA.

The primers’ sequences are as follows: IFN-β forward, 5′-CTCCTGGCTAATGTCTATCA-3′; IFN-β reverse, 5′-GCAGAATCCTCCCATAATAT-3′; GAPDH forward, 5′-GAAGGTGAAGGTCGGAGTC-3′; GAPDH reverse, 5′-GAAGATGGTGATGGGATTTC-3′.

### Immunofluorescence.

The cells were fixed with 4% paraformaldehyde for 15 min at room temperature, permeabilized cells were incubated with 0.5% Triton X-100 for 10 min, and cells were blocked with 1% bovine serum albumin (BSA) for 20 min. Cells were incubated with the corresponding primary and secondary antibodies, 4′,6-diamidino-2-phenylindole (DAPI) was added to stain nuclei, and then cells were stored in a refrigerator at 4°C and kept away from light. Observations were performed using an Olympus laser confocal microscope (Winooski, VT, USA).

### Proximity ligation assay.

Protein interactions were tested using the DuoLink proximity ligation assay (PLA) kit (DUO92008; Sigma). Thirty-six hours after overexpression of the VP3-HA plasmid, the described immunofluorescence fraction was fixed, permeabilized, and blocked with DuoLink Closure Solution liquid for 60 min at 37°C. Cells were incubated with the corresponding primary antibody diluted in DuoLink dilution buffer. After washing, cells were incubated with species-specific PLA probes (positive and negative) for 1 h at 37°C under hybridization conditions. Then ligase was added and incubated at 37°C for 30 min to ligate the hybridized oligonucleotides. Amplification polymerase was added to produce a ligated product extending from the oligonucleotide arm of the PLA probe. Nuclei were stained with Duolink in *in situ* mounting medium containing DAPI.

### Western blotting and coimmunoprecipitation.

For immunoblotting, cells were transfected with the corresponding plasmid. After 24 h, the supernatant was discarded, and the cells were washed three times with prechilled PBS, centrifuged at 6,000 rpm for 5 min. Sixty microliters of RIPA lysate were added to lyse the cells on ice for 5 min, and then the cells were boiled at 99°C for 5 to 10 min. The protein samples were separated on 10% or 15% SDS-PAGE and then transferred onto nitrocellulose membranes (catalog number 66485; Pall Corporation). The membrane was blocked with 5% skim milk for 1 h. The primary antibody was incubated overnight at 4°C, followed by a secondary antibody labeled with horseradish peroxidase (HRP). Imaging was conducted with the ChemiDoc XRS+ gel imaging system (Bio-Rad, USA) after adding the coloring solution.

For coimmunoprecipitation, cells were lysed with a lysis buffer containing protease inhibitor for 30 min on a shaker at 4°C. The supernatant was then collected at 8,000 rpm for 10 min. Half of the supernatant was used as input, and then the corresponding beads were added to the remaining supernatant. The samples were incubated for 6 h at low speed in a circular shaker at 4°C. The beads were then centrifuged at 7,000 rpm for 2 min and rinsed six times with PBS. Finally, 50 μL glycine, pH 2.4, was added to elute the target protein, which was centrifuged at 7,000 rpm for 2 min. The supernatant was taken as the target protein. The PBS used above was prechilled in advance, and the centrifugation was performed at 4°C.

### Computational modeling.

To predict the interactions between VP3 and IRF7, the crystal structure of VP3 (adopted from EV-D68 structure; PDB accession number 4WM7) and the AlphaFold2 predicted model of IFR7 were applied as inputs to HDOCK (32, 33). Top ranking models of VP3-IRF7 complexes were clustered and analyzed based on binding interface properties.

Two milliliters of lentivirus was added to 1 × 10^6^ RD cells, and 10 mg/mL of Polybrene was diluted 1,000 times. The complete medium was changed after 6 h. Puromycin medium was added for selection (1.1 μg/mL puromycin) after 24 h.

### Statistics.

All results were plotted with Image Lab and GraphPad Prism and are presented as mean ± standard deviation (SD) from at least three independent experiments. A statistically significant difference was determined using SPSS with a two-tailed Student’s unpaired *t* test (*, *P* < 0.05; **, *P* < 0.01; ***, *P* < 0.001; n.s., not significant).

### Data availability.

All of the data are fully available without any restriction.
