# Supplementary material for: Enterovirus D68 VP3 Targets the Interferon Regulatory Factor 7 To Inhibit Type I Interferon Response
Source: Microbiol Spectr. 2023 May 1;11(3):e04138-22. doi: 10.1128/spectrum.04138-22 (PMC10269600; doi:10.1128/spectrum.04138-22)
Supplement: Supplemental file 1 — Supplemental material. Download spectrum.04138-22-s0001.pdf, PDF file, 0.8 MB [file spectrum.04138-22-s0001.pdf]

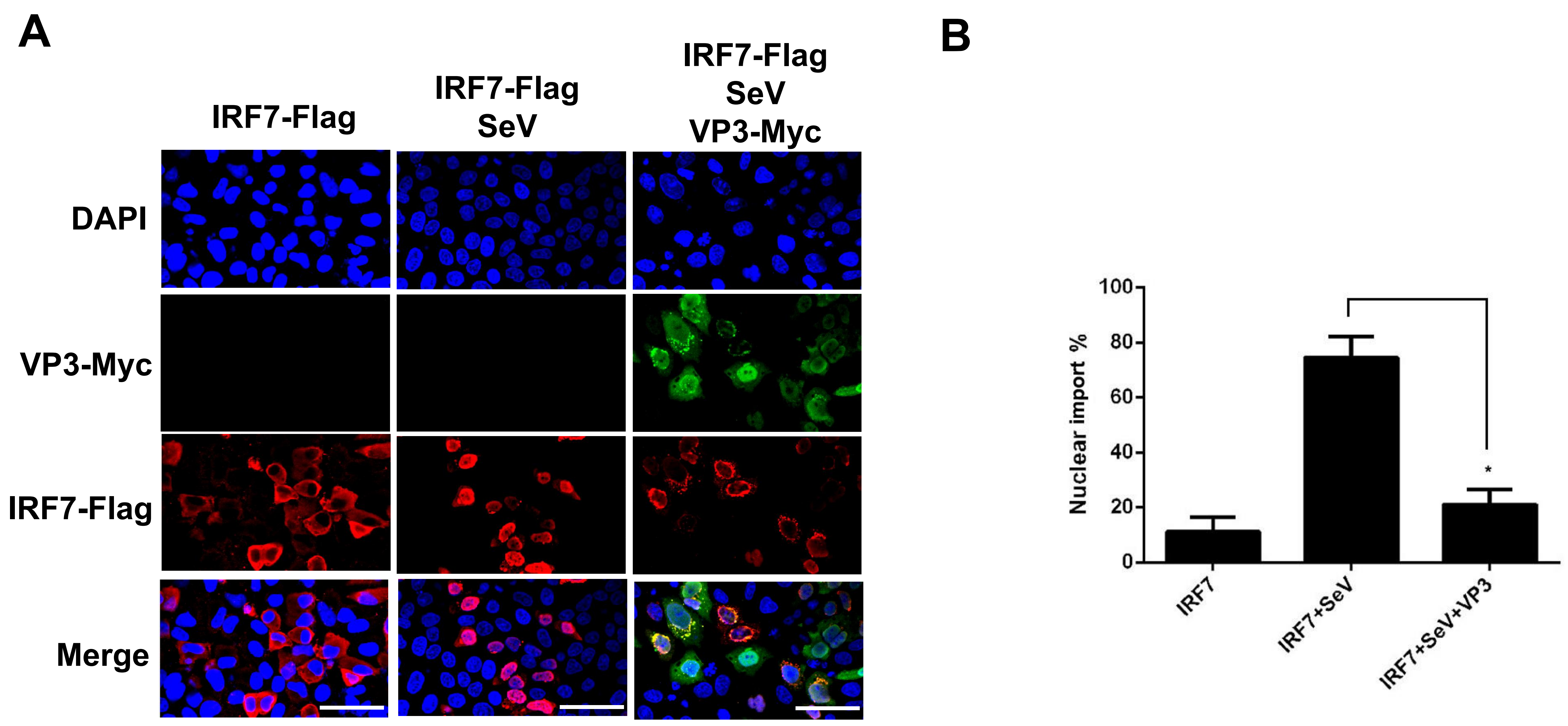

Fig S1

Fig S1 (A) EV-D68 VP3 inhibits the nuclear translocation of IRF7 after SeV stimulation in HeLa cells. HeLa cells were seeded on glass coverslips overnight and transfected with 300 ng IRF7-Flag or 500 ng VP3-Myc or both plasmids, 24 h after transfection, cells were infected with SeV (20 HA/mL) for 16 h. Then, the cells were analyzed using immunofluorescence staining with anti-Myc (green), anti-Flag (red), and DAPI (blue) and microscopy. (B) EV-D68 VP3 inhibits the nuclear translocation of IRF7 after SeV stimulation in HeLa cells. Co-localization of exogenous VP3 and IRF7 or IRF3 in HEK293T cells. HeLa cells were seeded on glass coverslips overnight and transfected with 300 ng IRF7/IRF3-Flag or 500 ng VP3-HA or both plasmids, 24 h after transfection, cells were infected with SeV for 16 h. Then, the cells were analyzed using immunofluorescence staining with anti-VP3 (green), antiIRF7 (red), and DAPI (blue) and microscopy. Two hundred cells were counted for calculating nuclear translocation for each group.

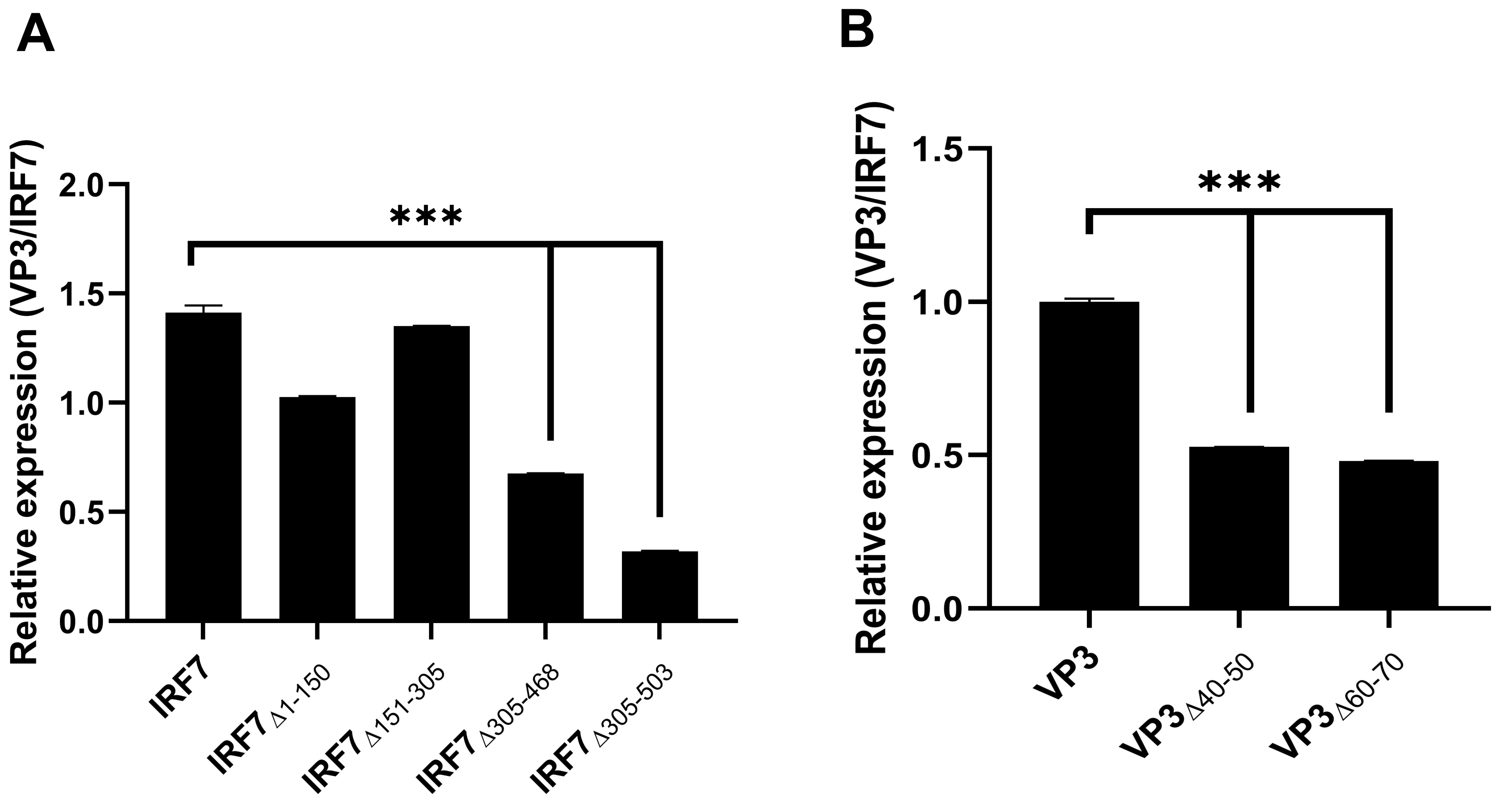

Fig S2

Fig S2. (A) Quantification of the relative expression of VP3/IRF7 in (Fig 5C). (B) Quantification of the relative expression of VP3/IRF7 in (Fig 5G).

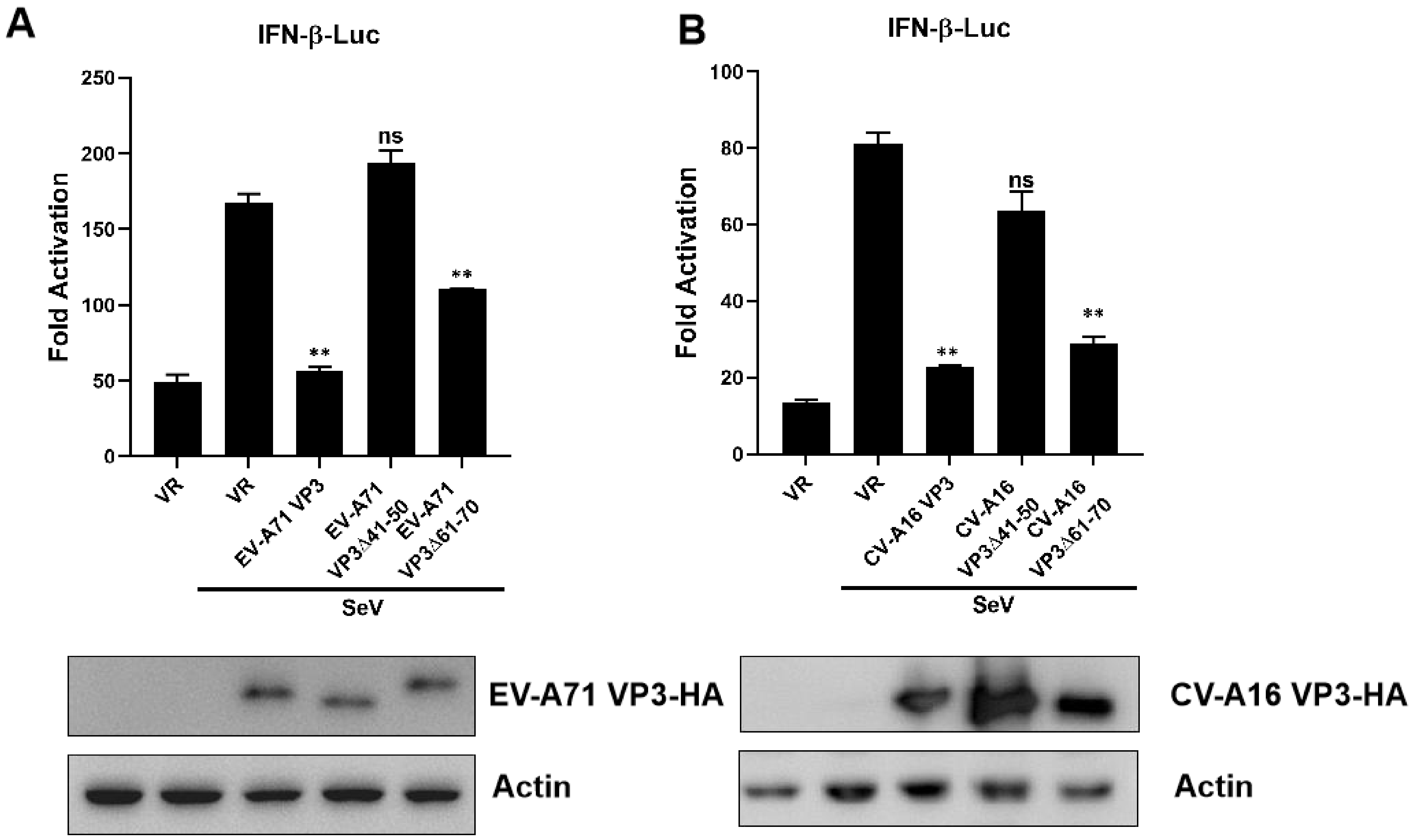

Fig S3

Fig S3. (A) HEK293T cells were transfected with 600ng EV-A71 VP3, EV-A71 VP3Δ41-50, EV-A71 VP3Δ61-70and 200 ng of IFN-β-Luc; 2 ng of pRL-SV40 was used as an internal control. Twenty-four hours after transfection, the cell lysates were subjected to a luciferase assay. (B) HEK293T cells were transfected with 600ng CV-A16 VP3, CV-A16 VP3Δ41-50, CV-A16 VP3Δ61-70 and 200 ng of IFN-β-Luc; 2 ng of pRL-SV40 was used as an internal control. Twenty-four hours after transfection, the cell lysates were subjected to a luciferase assay.

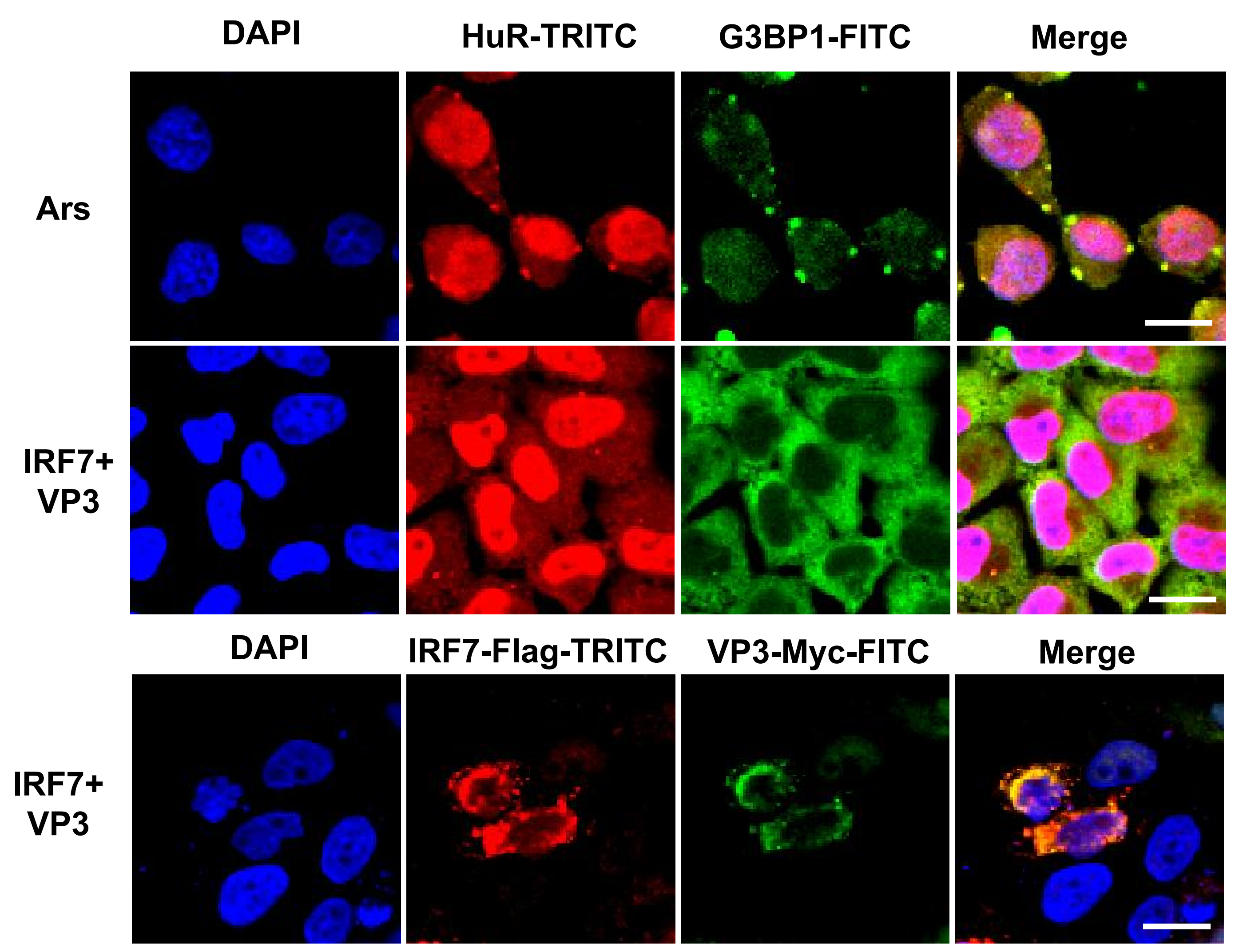

Fig S4

Fig S4. After VP3 and IRF7 were expressed together, SGs could not be produced.
